# Detection of Type VII collagen in odontogenic keratocyst: An immunohistochemical study

**DOI:** 10.4317/jced.54946

**Published:** 2019-04-01

**Authors:** Jochima-Eudora Cota, Anita Spadigam, Anita Dhupar

**Affiliations:** 1 Senior Resident, Department of Oral and Maxillofacial Pathology, Goa Dental College and Hospital, Bambolim, Goa; 2 Professor and Head of Department, Department of Oral and Maxillofacial Pathology, Goa Dental College and Hospital, Bambolim, Goa; 3 Professor, Department of Oral and Maxillofacial Pathology, Goa Dental College and Hospital, Bambolim, Goa

## Abstract

**Background:**

Separation of the epithelial lining from the underlying connective tissue wall has been a frequently observed and unique feature in odontogenic keratocysts (OKC), but not in other odontogenic cysts nor neoplasms. No study on OKC has been reported evaluating the role of type VII Collagen, the anchoring fibrils, which function in stabilising the epithelial structure. The purpose of this study was to assess the role of type VII collagen in the fragility of the epithelium leading to a high recurrence rate in OKCs.

**Material and Methods:**

Immunohistochemical staining with Abcam® Monoclonal Mouse Anti-Collagen VII Antibody [LH7.2] (used at a dilution of 1:200) on 30 tissues of OKC. The chi-square test was applied to confirm the statistical significance between the control and test groups. The frequencies of the pattern of distribution for the staining characteristics of collagen VII were calculated in the OKC samples.

**Results:**

Out of the 30 OKC samples 22 (73.3%) showed negative staining for type VII Collagen. Among the infected cases, 7 showed a positive basement membrane staining and one of the non-infected OKC showed positive basement membrane staining. However, none of the syndrome associated or recurrent OKCs showed any evidence of type VII collagen reactivity.

**Conclusions:**

Considering the distribution of type VII collagen in OKCs it can be concluded that type VII collagen expression is altered in OKCs, leading to destabilisation of the epithelium connective tissue interface thus rendering the epithelium-connective tissue interface fragile.

** Key words:**Type VII Collagen, Odontogenic Keratocyst, Basal Lamina, Immunohistochemistry.

## Introduction

Odontogenic keratocyst (OKC) is one of the most aggressive odontogenic cysts with a high recurrence rate of 5-62.5% (1), attributed to the presence of satellite cysts, tumorigenic potential inherent in remnants of dental lamina, basal cells of oral mucosa and the very thin, highly fragile nature of the epithelial cystic linings (2).

Separation of the epithelial lining from the underlying connective tissue wall is a frequently observed and unique feature in OKCs, but not in other odontogenic cysts (3) nor neoplasms (4). The pivotal role of the basement membrane has been demonstrated in certain diseases characterised by separation of the epithelium; however the expression of basement membrane components in OKCs has received little attention. Research has focussed on the distribution of collagen IV, laminin, fibronectin in various odontogenic cysts and tumours including OKCs (3,5-10). Various studies implicate the role of MMP 1, 2, 3, 7, 11, 12, 14, 16 and 26 (11-13), and TGF-beta (14,15) in the disengagement of the epithelium from the connective tissue. Philipsen *et al.* (16) demonstrated that deep to the lamina densa, the collagen shows signs of dissolution and often completely disappears, suggesting a collagen defect in OKCs. However, no study on OKCs has been reported evaluating the role of type VII Collagen, the anchoring fibrils, which function in stabilising the epithelial structure. Type VII collagen is synthesized by both keratinocytes and fibroblasts. It is composed of three identical alpha 1 chains that arrange as antiparallel dimers, which after proteolytic maturation assemble laterally to form anchoring fibrils. Type VII Collagen maintains the integrity of the epithelium by binding to Type I and Type III Collagen thorugh its interaction with laminin 332. Genetic deficiency of collagen VII causes dystrophic epidermolysis bullosa (DEB) (17).

Thus the purpose of this study was to assess the distribution of type VII collagen in the basal lamina complex of OKCs and to establish the role of type VII collagen in the fragility of the epithelium leading to a high recurrence rate in OKCs.

## Material and Methods

**Figure 1:**
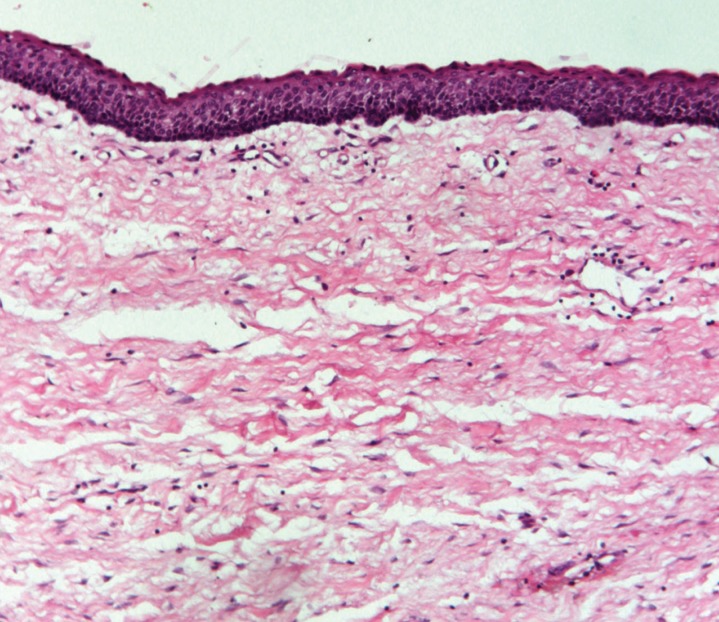
Haematoxylin and eosin stained section showing OKC (100X).

A retrospective study was conducted in the Department of Oral & Maxillofacial Pathology, Goa Dental College & Hospital on archival specimens, which were formalin fixed, processed, and paraffin embedded. In this study, a total of 30 previously diagnosed, hematoxylin and eosin stained histological specimens of OKCs (Fig. 1) were considered. The study also included 8 positive controls (normal skin and oral mucosa) and 2 negative controls (normal oral mucosa not subjected to primary antibody). No specific criteria in relation to age, sex, and the location within the oral cavity were applied to the chosen histological specimens. The samples were divided into non-infected OKCs (5 cases), infected OKCs (16 cases), syndrome associated OKCs (3 cases) and recurrent OKCs (6 cases).

Four micrometre thick sections were obtained from each of the selected cases for immunohistochemical staining with Abcam® Monoclonal Mouse Anti-Collagen VII Antibody [LH7.2] (used at a dilution of 1:200). The slides were deparaffinized by passing them through two changes of xylene for 5 min each. They were hydrated in two changes of 100% ethanol for 5 min each. The slides were then transferred to citrate buffer and enzymatic antigen retrieval using trypsin (0.05%, pH 7.8) was performed. After allowing to cool, they were washed in phosphate buffer solution. Immunohistochemical staining was then performed using DAKO Envision ™ kit. The slides were then mounted in DPX and observed under the light microscope for the results. Stained slides were examined under high power (40X) of the microscope.

The staining pattern of type VII Collagen was evaluated considering location and intensity of immunostaining as compared with the controls. The expression was graded according to its degree of intensity as absent (0), weak (1), moderate (2) and intense (3). The pattern of distribution at the basement membrane was evaluated as linear continuous, linear discontinuous, granular continuous, granular discontinuous and absent. The chi-square test was applied to confirm the statistical significance between the control and test groups ( Table 1). The frequencies of the pattern of distribution for the staining characteristics of collagen VII were calculated in the OKC samples ( Table 2). The data was analysed using Microsoft Excel 2010 and Statistical Package for Social Sciences (SPSS version 20).

**Table 1:**
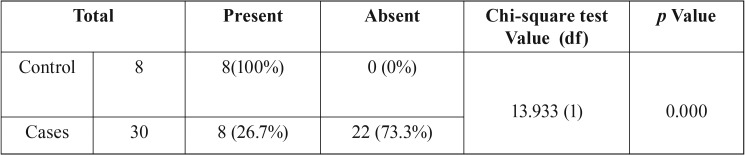
Cross tabulation (contingency table) of basement membrane staining in test and control groups.

**Table 2:**
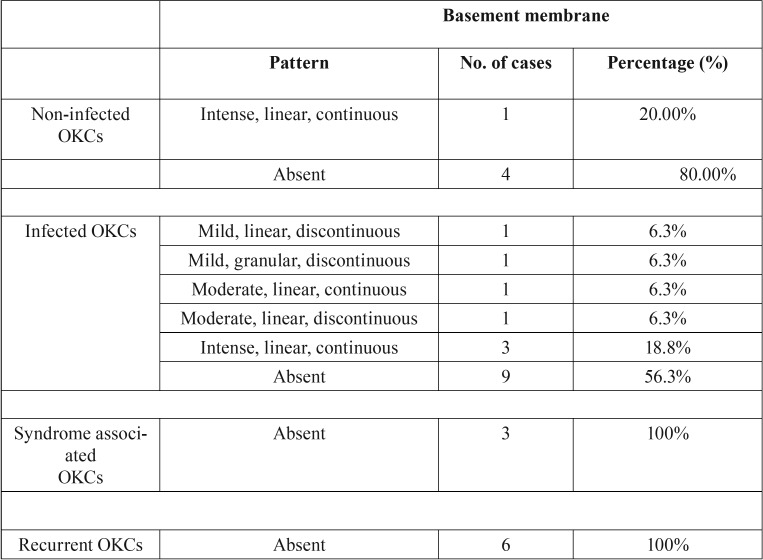
Frequency distribution of pattern of basement membrane staining in OKCs.

## Results

**Figure 2:**
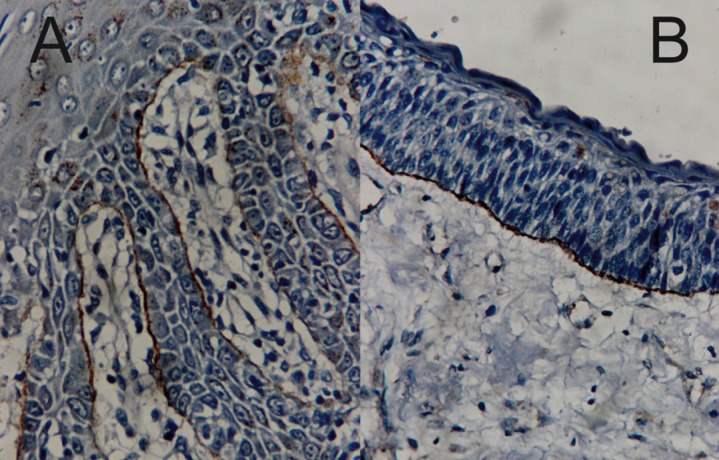
A) Positive control showing intense linear continuos staining of oral mucosa (100X). B) Non-infected OKC showing intense linear continuous basal lamina staining for type VII collagen (100X).

Out of the 30 OKC samples, 22 (73.3%) showed negative staining for type VII Collagen. A significant difference was found between the basement membrane staining seen in the OKCs and the normal control (*p*<0.05). Positive controls showed an intense linear staining (Fig. 2A). Among the infected cases, 7 showed a positive basement membrane staining and one of the non-infected OKC showed positive basement membrane staining (Fig. 2B). However, none of the syndrome associated or recurrent OKCs showed any evidence of type VII collagen reactivity. 4 cases showed intense linear continuous staining including the non-infected OKC. The other patterns seen were mild, linear, discontinuous; mild, granular, discontinuous; moderate, linear, continuous; moderate, linear, discontinuous.

**Figure 3:**
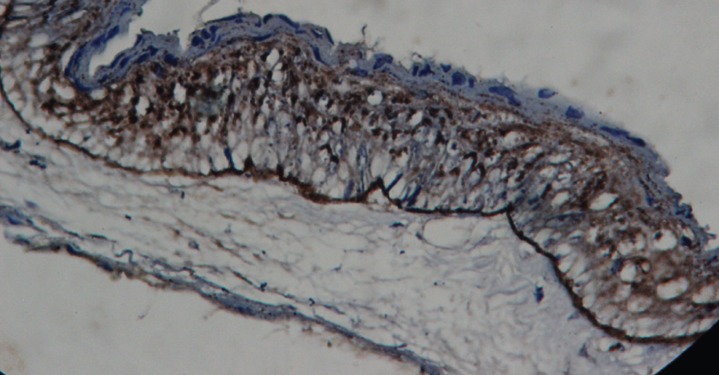
Non-infected OKC showing intense linear continuous basal lamina staining and intracytoplasmic retention for type VII collagen (100X).

The only positive non-infected OKC showed an intense intracytoplasmic granular basal cell staining (Fig. 3). Among the infected OKCs 4 showed a positive basilar intracytoplasmic staining and 5 cases showed a suprabasilar intracytoplasmic granular staining. None of the recurrent and syndrome associated OKCs showed any intracytoplasmic staining.

## Discussion

In the present study, type VII collagen staining was observed in the basement membrane region of OKCs ranging from incomplete staining with large or focal interruptions to staining being absent in some lesions; a complete staining without any focal or large interruptions was not observed in any of the cases. Type VII collagen has not been studied in odontogenic cysts and tumours till date. Hence, this study is a first of its kind.

Philipsen *et al.* (16) demonstrated that collagenolysis produced by a collagenase or other proteases deep to the lamina densa is responsible for the ready separation of OKC epithelium from its supporting capsule. Studies by Cavalcante RB *et al.* (12) found a higher expression of MMP-1, MMP-7 and MMP-26 in syndrome associated OKCs which could explain the increased OKC aggressiveness associated with Nevoid basal cell carcinoma syndrome. This is reflected in the findings of our study where none of the syndrome associated OKCs showed any immunoreactivity to collagen VII.

A positive collagen VII basement membrane staining was seen in 8 (26.7%) of the cases. Among the infected OKCs 7 showed a positive basement membrane staining, however among the non-infected only 1 showed a positive basement membrane staining. As demonstrated by König A *et al.* (15) TGF-β2 increases in a dose-dependent manner increasing the expression of collagen VII by cutaneous cells. Rudnicka *et al.* (14) suggested that the abundance of type VII collagen in the dermis of patients with systemic sclerosis may result from increased local expression of TGF-β3. Deyhimi P *et al.* (18) demonstrated a higher expression of TGF-β in OKCs than in OOCs (orthokeratinised odontogenic keratocyst). Zhong W *et al.* (19) found that TGF-β was highly expressed in OKCs.

Rodu *et al.* (20) demonstrated that 76% of OKCs exhibit marked inflammation within the connective tissue cyst wall and postulated that inflammation altered the biological behaviour of OKCs. Paula AMB *et al.* (21) suggested that growth factors and cytokines released within the fibrous tissue capsule of OKCs may be responsible for greater proliferative activity in inflamed OKCs compared to non-inflamed OKCs. While this study cannot prove the hypothesis that inflammation plays a role in aggressiveness of OKC, it can be postulated that inflammation causes an increase in the proliferative activity which alters the formation and secretion of type VII collagen.

Another peculiar finding in this study was the presence of a positive granular intracytoplasmic staining of the basal and suprabasal cells in our cases that show positive staining at the basement membrane. A similar finding was noted by Grewal HK *et al.* (6). They observed positive granular cytoplasmic staining of collagen IV within epithelial cells of OKC lining with an increase in intensity towards the superficial epithelial layers and in the corrugated parakeratinized layer. This may suggest a specific nature of the corrugated parakeratin and highlight its difference from the parakeratin laid down by the oral squamous epithelial cells.

Intracytoplasmic retention of type VII collagen was noted by Fine JD *et al.* (22) They demonstrated that the perinuclear “stellate bodies” in patients with transient bullous dermolysis of the new-born corresponds to type VII collagen suggesting the likelihood that such keratinocytes have a functional defect in transport of type VII collagen to the extracellular space or they may instead represent abnormal phagocytosis of the extracellular deposits of type VII collagen. Kainulainen T *et al.* (23) indicated that a high synthesis level, but an impaired distribution, of type VII collagen is highly characteristic of carcinoma cells in oral squamous cell carcinoma. Martins VL *et al.* (24) demonstrated that loss of type VII collagen in squamous cell carcinoma results in disordered epithelial terminal differentiation. Thus, correlating the studies it can be postulated that absence of type VII collagen and its intracytoplasmic accumulation can indicate the aggressiveness of OKCs.

Epithelial-mesenchymal interactions result in differentiation of epithelial cells into ameloblasts and mesenchymal cells into odontoblasts thus mediating tooth development. Umemoto *et al.* (25) suggested the importance of anchoring fibrils in the epithelial-mesenchymal interaction during tooth formation, especially in amelogenesis and ameloblast differentiation. Based on this, it can be speculated that type VII Collagen has a role in the pathogenesis of OKC, but this possibility has to be researched in future studies.

## Conclusions

Considering the distribution of type VII collagen in OKCs it can be concluded that type VII collagen expression is altered in OKCs, leading to destabilisation of the epithelium-connective tissue interface thus rendering the epithelium fragile. We hypothesize that this altered expression could be due to the increased expression of collagenases in the stroma of the OKCs. The possibility of a functional defect in the transport of type VII collagen to the extracellular space cannot be ruled out. The resultant fragility of the epithelium causes a high recurrence rate of the OKCs as part of the epithelium could be left behind following surgical treatment.

Acknowledgement: We would like to thank Colgate Palmolive (India) limited for the part sponsorship under their MDS research programme.
